# Asymptomatic Bacteriuria, antimicrobial susceptibility pattern and associated risk factors among pregnant women attending antenatal care in Assosa General Hospital, Western Ethiopia

**DOI:** 10.1186/s12866-021-02417-6

**Published:** 2021-12-16

**Authors:** Duresa Abu, Teferra Abula, Tesfu Zewdu, Muluken Berhanu, Tamiru Sahilu

**Affiliations:** 1 Department of Pharmacy, College of Health Science, Assosa University, Assosa, Ethiopia; 2 Department of Pharmacology and Clinical Pharmacy, Addis Ababa University, Addis Ababa, Ethiopia; 3 Department of Nursing, College of Health Science, Assosa University, Assosa, Ethiopia; 4 College of Health Science, Assosa University, Assosa, Ethiopia

**Keywords:** Asymptomatic Bacteriuria, Antimicrobial susceptibility, Pregnant women, Antenatal care

## Abstract

**Background:**

Asymptomatic bacteriuria is a common problem in pregnant women and about 40% of women with untreated asymptomatic bacteriuria during pregnancy develop pyelonephritis, which might lead to low birth weight, premature rupture of membranes, and preterm labour. Therefore, this study aimed to assess the prevalence of asymptomatic bacteriuria, antimicrobial susceptibility pattern of the isolates among pregnant women attending the antenatal care of Assosa general hospital, western Ethiopia.

**Methods:**

A facility-based cross-sectional study was conducted from January to February 2019. Two hundred and eighty-three pregnant women with no symptoms of urinary tract infections participated in the study. Bacterial isolates were identified as per the standard bacteriological procedure using colony characteristics, Gram-staining, and series of biochemical tests. Antimicrobial susceptibility test was carried out by Kirby- Bauer disk diffusion technique on Muller-Hinton agar medium and the diameter of zone of inhibition was interpreted according to Clinical Laboratory Standard Institute guidelines.

**Results:**

The overall prevalence of asymptomatic bacteriuria among pregnant women in this study was 13.78% (i.e. 39 out of 283 urine samples were positive for bacterial isolates). *E. coli* was the most predominant isolate (53.8%) followed by *K. pneumoniae* (17.95%), *S. aureus* (15.4%), and *coagulase-negative staphylococci* (12.8%). Gram-negative bacteria were highly resistant to tetracycline (96.4%), and ampicillin (90.5%).

**Conclusion:**

Significant bacteriuria was observed in asymptomatic pregnant women. A large number of the bacterial isolates were resistant to the commonly used antimicrobial drugs.

## Background

Asymptomatic bacteriuria (ASB) is defined as the presence of 10^5^ and more colony-forming units (CFU) per milliliter (mL) of urine in the absence of specific symptoms of acute urinary tract infections (UTIs) [1, 2]. Pregnant women are at increased risk of asymptomatic bacteriuria due to mechanical factors, hormonal changes, urinary stasis, and reflux of urine from the bladder to the ureters [3]. Therefore, screening for bacteriuria during pregnancy irrespective of whether a patient is symptomatic or not is important in the first care setting as early treatment can prevent subsequent complications [4, 5]. Both Gram-negative and -positive bacteria are predominantly responsible for ASB during pregnancy [1]. Screening for asymptomatic bacteriuria became standard obstetric care, and most antenatal guidelines today include routine screening for asymptomatic bacteriuria. The United States preventive services task force strongly recommends screening and treatment, and similar recommendations are included in guidelines from the Infectious Diseases Society of America, the National Institute for Clinical Excellence, the European Association of Urology, the Canadian Task Force on Preventive Care, and most recently from the Scottish Intercollegiate Guidelines Network [6–11]. Even if Standard Treatment Guidelines in Ethiopia recommend screening and treatment of ASB, unfortunately, screening and treatment of pregnant women for asymptomatic bacteriuria never became standard ANC follow-up practice in Ethiopia. In most developing countries including Ethiopia, the limited health care budgets and lack of adequate laboratory facilities or trained microbiologists or both affect the health care system a lot [12, 13]. Antimicrobial drug resistance in bacteriuria is increasing worldwide and some bacteria are virulent and capable of acquiring multidrug resistance to antimicrobials. Rates of antimicrobial resistance vary according to geographic locations and they are directly proportional to the use and misuse of antimicrobials. However, there was a scarcity of data on the prevalence of ASB and antimicrobial susceptibility of the bacterial isolates among pregnant women in the Benishangul Gumuz Region, Western Ethiopia. Therefore, this study was aimed to assess the prevalence of asymptomatic bacteriuria, antimicrobial susceptibility pattern of the bacterial isolates and related risk factors among pregnant women attending antenatal care (ANC) of Assosa General Hospital, Western Ethiopia.

## Materials and methods

This study was conducted at Assosa General Hospital from January to February 2019. The hospital is found in Assosa town and the town is 670 km away from Addis Ababa, the capital city of Ethiopia. All pregnant women attending the ANC of Assosa General Hospital for ANC services and didn’t present signs and symptoms of UTIs were included in the study.

### Study design

A facility-based cross-sectional study was conducted among pregnant women attending antenatal (ANC) of Assosa General Hospital, Benishangul Gumuz Region, Western, Ethiopia.

### Source population

All pregnant women attending ANC of Assosa General Hospital for ANC services.

### Study population

All pregnant women attending ANC of Assosa General Hospital during the study period and fulfilled the inclusion criteria.

#### Inclusion criteria

All pregnant women who attended ANC of Assosa General Hospital for ANC services during the study period, women with asymptomatic bacteriuria, and willing to participate in the study were included.

#### Exclusion criteria

Pregnant women with a history of antibiotic therapy in the previous 2 weeks and who were critically sick and unable to answer the questionnaire were excluded.

### Bacterial culture, identification, and antimicrobial susceptibility testing

After taking written informed consent from the pregnant women, about 5 mL of freshly voided midstream urine samples were collected using a sterile screw-capped, wide-mouth container. The pregnant women wash their hands, cleanse the genitals with clean water, and collect the middle urine into a wide-mouthed urine container. The urine specimens obtained from the pregnant women were directly inoculated on cystine lactose electrolyte deficient agar (CLED) (Oxoid, Ltd., England) media by streak plate method using calibrated inoculating wire loop (0.001 mL). The urine samples’ culture plates were incubated in the aerobic environment at 37 °C for 24-48 h and the plates were checked for the growth of pathogens. All plates with 10^5^ and more bacterial colonies per milliliter (ml) of urine were sub-cultured onto MacConkey agar (Oxoid, England), and 5% sheep blood agar (Oxoid, England) for further identification. Bacterial isolates were identified as per the standard bacteriological procedure using colony characteristics, Gram-staining, and a series of biochemical tests (such as Kligler’s Iron Agar (KIA), Sulphur Indole Motility (SIM) media, citrate, oxidase, urease, and catalase, and coagulase). The antimicrobial susceptibility test was carried out by Kirby- Bauer disk diffusion technique on Muller-Hinton agar medium and the diameter of the zone of inhibition was interpreted according to Clinical Laboratory Standard Institute (CLSI) 2017 guideline [14].

### Data quality assurance

A detailed quality assurance procedure was used to keep the quality of data. To avoid language barriers and ambiguities, the questionnaire was first translated to the local language and 5% of the questionnaire was pre-tested. The training was given to data collectors to minimize technical errors and to maintain the quality of data. The collected data were checked for completeness at the end of each day of data collection. Standard operating procedures (SOPs) were strictly followed during all aspects of laboratory procedure including sample collection, sample inoculation, culturing, biochemical tests, and antimicrobial susceptibility testing. The culture media was tested for sterility and performance. The American Type Culture Collection (ATCC) reference strains such as *Escherichia coli* ATCC® 25922, *Escherichia coli* ATCC® 35218 (when testing amoxicillin-clavulanic acid), and *Staphylococcus aureus* ATCC® 25923 were used as quality control parameters during culture and antimicrobial susceptibility tests. All the standard strains were obtained from the Ethiopian Public Health Institute.

### Data processing and analysis

Data was initially entered and cleaned using Epi-data version 3.1 and exported to SPSS version 20.0 for analysis. Statistical analysis was performed using SPSS and it was summarized and presented by frequency tables and summary statistics presented by graphs, tables, and narratives. Descriptive statistics were done to indicate the frequency of the variables and multiple logistic regression analysis was used to determine the predictors of asymptomatic bacteriuria. *P*-value < 0.05 was considered statistically significant.

### Ethical consideration

The study was ethically approved by the Ethical review board (ERB) of Addis Ababa University College of Health Sciences, School of pharmacy (ERB/SOP/42/11/2018). Official permission was obtained from Benishangul Gumuz Region Health Bureau and Assosa General Hospital administrative bodies. During data collection, each study participant was informed about the purpose of the study and written informed consent was obtained from the pregnant women. Anyone who was not willing to participate in the study was excluded from the study. Any information concerning the study participants was kept confidential and the specimen collected from the study participants was only analyzed for the intended purposes. Pregnant women who had significant bacteriuria received appropriate treatment according to the national guideline.

### Operational definitions

**Asymptomatic bacteriuria (ASB):** It is the presence of significant bacteria (≥ 10^5^cfu/ml) in a patient without signs or symptoms of UTI.

**Midstream urine:** A specimen obtained from the middle part of urine flow.

**Multidrug resistance:** is antimicrobial resistance shown by a species of microorganism to three or more antibiotics of different classes.

## Results and discussion

**Fig. 1:**
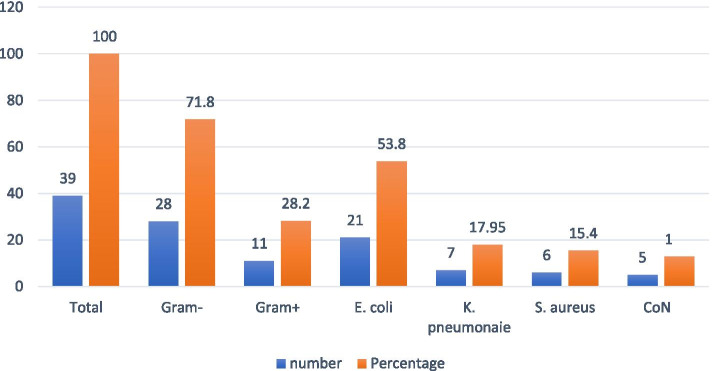
Frequency of bacterial uropathogens isolated from pregnant women

A total of 283 pregnant women without signs and symptoms of UTI were included in this study. The prevalence of ASB among pregnant women in this study was 13.78% (95%CI: 9.8–18.8%) (*n* = 39/283). As it was shown in Fig. 1, Gram-negative bacteria, 9.89% (95%CI: 6.6–14.3%) were more prevalent than Gram-positive bacteria. The predominance of Gram-negative bacteria was also reported in studies conducted in Adigrat, Northern Ethiopia (64.7%) [15], Nairobi, Kenya (64.1%) [16], the central region of Iran (69.6%) [17], and Bengal India (62.3%) [18]. This might be due to the proximity of the female urethra to the anal area. Besides, difficulties during pregnancy in cleaning the genital area after defecation might result in contamination of the female urinary tract with fecal bacteria (mostly Gram-negative). However, in contrast to our findings, the predominance of Gram-positive bacteria was reported in other studies done in Dessie, Northeast Ethiopia [19], and Hawassa, Southern Ethiopia [20]. The possible explanation for this discrepancy might be due to differences in environmental conditions (temperature, humidity, etc), and the level of antimicrobial usage by the patients, in these study sites, which could affect the distribution of bacteria in different regions in the same country. *E. coli* (53.8%) was the predominant bacterial isolate observed in this study followed by *K. pneumonia* (17.95%), *S. aureus* (15.4%), and *CoNS* (12.8%). The predominance of *E. coli* in our study is consistent with studies done in Bahir Dar, Northwest Ethiopia [21], Hawassa, Southern Ethiopia [22], Ghana [23], Egypt [24], and India [19]. The acquired ability of *E. coli* to produce several virulence factors that facilitate colonization and invasion of the urinary epithelium might be one possible explanation for the predominance of *E. coli* in pregnant women with ASB [25].

Regarding the antimicrobial susceptibility pattern of bacterial uropathogens, our findings showed that bacterial uropathogens isolated from pregnant women with asymptomatic UTI develop resistance to commonly used antimicrobial agents. Most of the Gram-negative bacterial isolates were susceptible to meropenem (96.4%), ceftazidime (85.7%), whereas most were resistant to tetracycline (96.4%), and ampicillin (90.5%) (Table 1). Our finding is in line with studies done in Dessie, Northeast Ethiopia [25], Baghdad, Iraq [26], and Kanpur, India [27]. However, contrary to studies done in Kashmir [28], and Adigrat, Northern Ethiopia [29]. The easy accessibility of the commonly prescribed antimicrobials over-the-counter combined with the misuse of the antibiotics by both the patients and clinicians, lack of trained personnel for urine culture, and frequent use of common antimicrobial agents without prescription might be responsible for the observed high prevalence of antimicrobial resistance to commonly used antibiotics. Increased resistance of *E. coli* to β-lactamase inhibitors and emergence of resistance (4.8%) to meropenem that is active against extended-spectrum β-lactamase (ESBL) positive strains in this study area is a worrying situation that needs continuous monitoring and surveillance of antimicrobial resistance of *E. coli* mainly in highly vulnerable groups such as pregnant women.

**Table 1:** Antimicrobial susceptibility pattern of Gram-negative bacteria isolated from the urine of pregnant women without signs and symptoms of UTI attending ANC in Assosa General Hospital

Bacterial isolates (no.)	Antimicrobial agents tested													
		TER	NA	CIP	SMT	GEN	AMK	TOB	AMP	AMX-C	CEF	CTX	CAZ	MER
***E. coli*** **(*n*** **= 21)**	S; n, (%)	1 (4.8)	9 (42.9)	14 (66.6)	5 (23.8)	10 (47.6)	18 (85.7)	21 (100)	2 (9.5)	5 (23.8)	15 (71.4)	16 (76.2)	17 (81)	20 (95.2)
	R; n, (%)	20 (95.2)	12 (57.1)	7 (33.4)	16 (76.2)	11 (52.4)	3 (14.3)	0	19 (90.5)	16 (76.2)	6 (28.6)	5 (23.8)	4 (19)	1 (4.8)
***K. pneumonia*** **(7)**	S; n, (%)	0	2 (28.6)	2 (28.6)	0	2 (28.6)	3 (42.9)	3 (42.9)	–	2 (28.6)	3 (42.9)	2 (28.6)	7 (100)	7 (100)
	R; n, (%)	7 (100)	5 (71.4)	5 (71.4)	7 (100)	5 (71.4)	4 (57.1)	4 (57.1)	–	5 (71.4)	4 (57.1)	5 (71.4)	0	0
**Total (*n*** **= 28)**	S; n, (%)	1 (3.6)	11 (39.3)	16 (57.1)	5 (17.9)	12 (42.9)	21 (75)	24 (85.7)	2 (9.5)	7 (25)	18 (64.3)	18 (64.3)	24 (85.7)	27 (96.4)
	R; n, (%)	27 (96.4)	17 (60.7)	12 (42.9)	23 (82.1)	16 (57.1)	7 (25)	4 (14.3)	19 (90.5)	21 (75)	10 (35.7)	10 (35.7)	4 (14.3)	1 (3.6)

*S* susceptible; *R* resistant; *CIP* ciprofloxacin; *CTX* Cefotaxime; *TER* tetracycline; *SMT* sulfamethoxazole-trimethoprim; *CEF* ceftriaxone; *AMP* ampicillin; *Amx-c* amoxicillin-clavulanic acid; *CAZ* ceftazidime; *GEN* gentamycin; *AMK* amikacin; *MER* meropenem; NA: nalidixic acid; *TOB* Tobramycin

The finding of our study also showed that Gram-positive bacterial isolates were highly resistant to tetracycline (100%), trimethoprim-sulfamethoxazole (81.8%), penicillin (72.72%), and nalidixic acid (54.5%) (Table 2**)**. Relatively similar resistance rates of Gram-positive isolates for these antibiotics were also reported from studies done in Gondar, Northwest Ethiopia [30], and India [19] which might be due to the indiscriminate and misuse of the antibiotics for empirical therapy. In the current study, most Gram-positive bacteria isolates were susceptible to vancomycin (100%, we are unsure of one of obtained result and we excluded this strain from AST analysis), clindamycin (90.9%), chloramphenicol (81.8%), norfloxacin (72.7%), ciprofloxacin (63.6%), and erythromycin (63.6%). A relatively similar susceptibility pattern of Gram-positive isolates to most of these antimicrobial agents is reported from studies done in Adama, Ethiopia [16].

**Table 2:** Antimicrobial susceptibility pattern of Gram-positive bacteria isolated from the urine of pregnant women without signs and symptoms of UTI attending ANC in Assosa General Hospital

Bacterial isolates (n)	Antimicrobial agents tested												
		CLI	ERY	CAF	PEN	CAZ	NA	TOB	CIP	STX	NOR	VAN	TET
***S. aureus*** **(6)**	S; n (%)	6 (100)	4 (66.7)	5 (83.3)	1 (16.7)	–	3 (50)	6 (100)	4 (66.7)	1 (16.7)	5 (83.3)	5 (100)^a^	0
	R	0	2 (33.3)	1 (16.7)	5 (83.3)	–	3 (50)	0	2 (33.3)	5 (83.3)	1 (16.7)	0	6 (100)
***CoNS*** **(5)**	S; n (%)	4 (80)	3 (60)	4 (80)	2 (40)	–	2 (40)	4 (80)	3 (60)	1 (20)	3 (60)	5 (100)	0
	R; n (%)	1 (20)	2 (40)	1 (20)	3 (60)	–	3 (60)	1 (20)	2 (40)	4 (80)	2 (40)	0	5 (100)
**TOTAL (11)**	S; n (%)	10 (90.9)	7 (63.64)	9 (81.81)	3 (27.27)		5 (45.45)	10 (90.9)	7 (63.64)	2 (18.18)	8 (72.72)	10 (90.9)	0
	R; n (%)	1 (9.1)	4 (36.36)	2 (18.18)	8 (72.72)		6 (54.54)	1 (9.1)	4 (36.36)	9 (81.81)	3 (27.27)	0	11 (100)

*CLI* clindamycin; *ERY* erythromycin; *CAF* Chloramphenicol; *Pen* penicillin; *CAZ* ceftazidime; *CIP* ciprofloxacin; *TET* tetracycline; *STX* trimethoprim-sulfamethoxazole; *NA* nalidixic acid; *TOB* Tobramycin; *NOR* norfloxacin; *VAN* vancomycin; *CoNS Coagulase Negative Staphylococci*

^a^ We are unsure of one of obtained result and we excluded this strain from AST analysis; so, we obtained 100% *S. aureus* strains susceptible to vancomycin

In this study, multi-drug resistance (MDR) was seen in 74.4% of the isolated bacterial uropathogens (Table 3**)**. Almost a similar trend was reported from Dessie, Northeast Ethiopia (72.4%) [20], and Tikur Anbessa Specialized Hospital, Addis Ababa (74%) [31]. Self-medication and non-compliance of antimicrobial drugs might contribute to rapid drug resistance.

**Table 3:** Multidrug resistance (MDR) pattern of the isolated bacterial uropathogens among pregnant women attending ANC in Assosa General Hospital

Bacterial isolate		Antimicrobial pattern, n (%)						
		Total (%)	R0	R1	R2	R3	R4	R5
**Gram- negative**	Total; n (%)	28 (100)	1 (3.6)	1 (3.6)	5 (17.86)	0	4 (14.3)	17 (60.7)
	*Escherichia coli*; n (%)	21 (75)	1 (100)	1 (100)	3 (60)	0	4 (100)	12 (70.6)
	*K. pneumonia*; n (%)	7 (25)	0	0	2 (40)	0	0	5 (29.4)
**Gram-positive**	Total; n (%)	11 (100)	0	2 (18.18)	1 (9.1)	2 (18.18)	2 (18.18)	4 (36.36)
	*Staphylococcus aureus*; n (%)	6 (54.54)	0	1 (50)	0	2 (100)	1 (50)	2 (50)
	*C0NS*; n (%)	5 (45.45)	0	1 (50)	1 (100)	0	1 (50)	2 (50)
**TOTAL**		39 (100)	1 (2.6)	3 (7.7)	6 (15.4)	2 (5.13)	6 (15.4)	21 (53.85)

*R0* no resistance; *R1* resistance to one; *R2* resistance to two; *R3* resistance to three; *R4* resistance to four; *R5* resistance to five antibiotics, *CoNS* Coagulase Negative Staphylococci

In our study, educational status (uneducated, read and write), gestational age (1st and 2nd trimester), and history of UTI were significantly associated with asymptomatic bacteriuria among pregnant women. Asymptomatic bacteriuria typically occurs during early pregnancy [32]. Similarly, gestational period (1st trimester and 2nd trimester) [33], history of UTI, and educational status [34] association with asymptomatic bacteriuria were reported in the previous studies.

### Factors associated with the asymptomatic bacteriuria

**Fig. 2:**
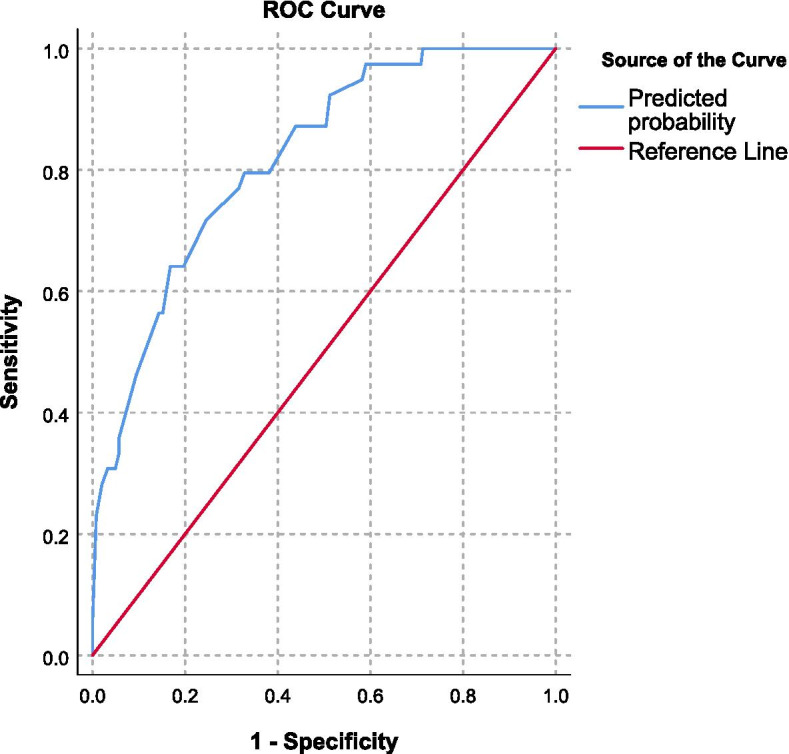
Receiver operating characteristic (ROC) curve for the Factors associated with the ASB. The area under the curve (AUC) was 81.8% (95% CI, 75.2–88.5), indicating the discriminating ability of the model is an excellent performance

**Fig. 3:**
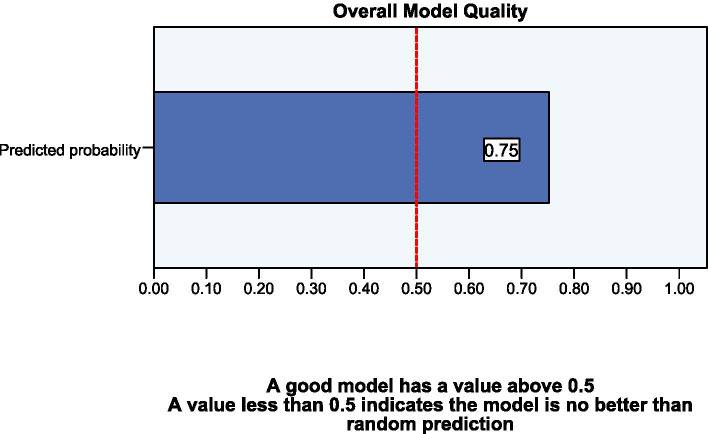
Overall model quality

In multivariate logistic regression, educational status (uneducated, read and write), gestational age (1st and 2nd trimester), and history of UTI were significantly associated with asymptomatic bacteriuria among pregnant women. Pregnant women with educational status of only being able to read and write and uneducated had higher odds of experiencing asymptomatic bacteriuria compared to pregnant women with educational status of higher education. Pregnant women with the gestational age of 1st and 2nd trimester were more likely to experience asymptomatic bacteriuria compared to pregnant women with the gestational age of 3rd trimester. Pregnant women without a history of UTI were less likely to experience asymptomatic bacteriuria compared to pregnant women with a history of UTI (Table 4). The receiver operating characteristic (ROC) curve for the factors associated with the ASB showed the area under the curve (AUC) was 81.8% (95% CI: 75.2–88.5), indicating the discriminating ability of the model is an excellent performance (Fig. 2). The overall model quality is depicted in Fig. 3.

**Table 4:** Factors associated with asymptomatic bacteriuria among pregnant women attending ANC in Assosa General Hospital

Variables		ASB		Total n (%)	COR (95% CI)	P value	AOR (95% CI)	P value
		Negative; n (%)	Positive; n (%)					
Residence	Rural	39 (13.8%)	3 (1.1%)	42 (14.8%)	1		1	
	Urban	205 (72.4%)	36 (12.7%)	241 (85.2%)	2.28 (0.67–7.784)	0.187	3.61 (0.864–15.082)	0.079
Educational status	higher and above	113 (39.9%)	8 (2.8%)	121 (42.8%)	1		1	
	Uneducated	23 (8.1%)	12 (4.2%)	35 (12.4%)	7.37 (2.709–20.046)	0.000	13.586 (4.174–44.224)	0.000
	read and write	21 (7.4%)	8 (2.8%)	29 (10.2%)	5.381 (1.818–15.925)	0.002	6.699 (2.1–21.368)	0.001
	primary school	87 (30.7%)	11 (3.9%)	98 (34.6%)	1.786 (0.689–4.63)	0.233	2.024 (0.757–5.417)	0.16
Gestational age	3rd trimester	90 (31.8%)	4 (1.4%)	94 (33.2%)	1		1	
	1st trimester	60 (21.2%)	13 (4.6%)	73 (25.8%)	4.875 (1.517–15.665)	0.008	5.503 (1.597–18.956)	0.007
	2nd trimester	94 (33.2%)	22 (7.8%)	116 (41%)	5.266 (1.746–15.88)	0.003	5.272 (1.655–16.795)	0.005
History of UTI	Yes	114 (40.3%)	29 (10.2%)	143 (50.5%)	1		1	
	No	130 (45.9%)	10 (3.5%)	140 (49.5%)	0.302 (0.141–0.648)	0.002	0.285 (0.125–0.65)	0.003

*ASB* asymptomatic bacteriuria; *AOR* Adjusted odds ratio; *COR* Crude odds ratio; *UTI* Urinary tract infection

## Conclusion

A considerable percentage of pregnant women without UTI had asymptomatic bacteriuria. *E. coli* was the most predominant bacterial isolate followed by *K. pneumoniae*, *S. aureus*, and *CoNS*. A large number of the bacterial isolates were resistant to the commonly used antimicrobial drugs. There was a high prevalence of MDR bacterial isolates among pregnant women with ASB in this study area.

## Data Availability

The datasets generated and/or analyzed during the current study are available in the Research Square repository, 10.21203/rs.3.rs-138813/v1, and 10.21203/rs.3.rs-100055/v1

## Abbreviations

AMR: Antimicrobial resistance
ANC: Antenatal Care
ASB: Asymptomatic Bacteriuria
AST: Antimicrobial Susceptibility Test
CFU: Colony Forming Unit
*CoNS*: *Coagulase Negative Staphylococci*
CI: Confidence Interval
CLED: Cysteine Lactose Electrolyte Deficient
CLSI: Clinical Laboratory Standard Institute
*E. coli*: *Escherichia Coli*
MDR: Multidrug resistance
MHA: Muller Hinton Agar
NCCLS: National Committee for Clinical Laboratory Standard
OR: Odds Ratio
RPM: Revolutions per Minute
ROC: receiver operating characteristic
UTI: Urinary Tract Infection
